# Supplementary material for: Mycoplasma genitalium Provokes Seminal Inflammation among Infertile Males
Source: Int J Mol Sci. 2021 Dec 15;22(24):13467. doi: 10.3390/ijms222413467 (PMC8707260; doi:10.3390/ijms222413467)
Supplement: Supplementary file 1 [file ijms-22-13467-s001.zip › ijms-1498523-supplementary.pdf]

Supplementary Tables and Figures

Supplementary Table S1. The distribution of patients (both unselected fertile and infertile groups, without exclusion of patients for hormonal, testicular volume and infertility causal factors) according to the presence or absence of an inflammatory reaction in semen (i. e. concentration of neutrophils) using different cut-off levels.

STI status of patients	Concentration of neutrophils semen ≥ 1.0 *M/mL*		Concentration of neutrophils semen ≥ 0.5 *M/mL*		Concentration of neutrophils semen ≥ 0.2 *M/mL*	
	Inflammatory reaction in semen:		Inflammatory reaction in semen:		Inflammatory reaction in semen:	
	Absent	Present	Absent	Present	Absent	Present
**STI negatives (N = 2193) [α]**	1988 (90.7%) [A]	205 (9.3%) [A]	1792 (81.7%) [B]	401 (18.3%) [B]	1358 (61.9%) [C]	835 (38.1%) [C]
***C. trachomatis* positives (N = 28)**	22 (78.6%) [D]	6 (21.4%) [D]	18 (64.3%)	10 (35.7%)	11 (39.3%)	17 (60.7%)
***M. genitalium* positives (N = 22)**	14 (63.6%)	8 (36.4%)	10 (45.5%)	12 (54.5%)	7 (31.8%)	15 (68.2%)

Annotations to Supplementary Table S1.

Abbreviations: STI – sexually transmitted infection

[α] Data about inflammatory reaction in semen is not available for 5 STI negative patients.

[A, B, C, D] p–value <0.005, Chi–squared test.

Supplementary Table S2. The detailed information with references for additional elimination criteria.

**(I) Defined causal factors of male infertility** (according to Punab et al [1]):
(1) genetic causes,<br>(2) secondary hypogonadism,<br>(3) congenital anomalies: systemic and/or in uro–genital tract,<br>(4) serious sexual dysfunctions,<br>(5) oncological diseases,<br>(6) seminal tract obstruction,<br>(7) other testicular factors<br>A. Acquired testicular damage (TD):<br>1. Exposure to high dose radiation in Chernobyl<br>2. Testis trauma with volume change<br>3. Mumps orchitis<br>4. Orchitis, epididymitis<br>5. Testicular torsion<br>6. Hernia operation with ipsilateral TD<br>7. Epididymal cyst operation with ipsilateral TD<br>8. Hydrocele operation with ipsilateral TD<br>9. Other testis operation with ipsilateral TD<br>B. Secondary testicular damage:<br>1. Anabolic steroids;<br>2. Medication – salasopyrin, trexan;<br>3. Status diagnosed post kidney transplantation
**(II) Bitesticular volume < 30 ml for unexplained reason**
**(III) Hormonal changes:**
(1) FSH ≥8.0 U/L [1, 2, 3, 4, 5]<br>(2) and/or LH >9.4 U/L [6]<br>(3) and/or FSH <1.0 U/L [1]<br>(4) and/or LH <1.0 U/L [1]<br>(5) and/or total testosterone <10.5 nmol/L [6]

Annotations to Supplementary Table S2.

References:

- [1] Punab, M., Poolamets, O., Paju, P., Vihljajev, V., Pomm, K., Ladva, R., Korrovits, P., & Laan, M. (2017). Causes of male infertility: a 9-year prospective monocentre study on 1737 patients with reduced total sperm counts. *Human reproduction (Oxford, England), 32*(1), 18–31. https://doi.org/10.1093/humrep/dew284
- [2] Bergmann, M., Behre, H. M., & Nieschlag, E. (1994). Serum FSH and testicular morphology in male infertility. *Clinical endocrinology, 40*(1), 133–136. https://doi.org/10.1111/j.1365-2265.1994.tb02455.x
- [3] Jensen, T. K., Andersson, A. M., Hjollund, N. H., Scheike, T., Kolstad, H., Giwercman, A., Henriksen, T. B., Ernst, E., Bonde, J. P., Olsen, J., McNeilly, A., Groome, N. P., & Skakkebaek, N. E. (1997). Inhibin B as a serum marker of spermatogenesis: correlation to differences in sperm concentration and follicle-stimulating hormone levels. A study of 349 Danish men. *The Journal of clinical endocrinology and metabolism, 82*(12), 4059–4063. https://doi.org/10.1210/jcem.82.12.4456
- [4] Sikaris, K., McLachlan, R. I., Kazlauskas, R., de Kretser, D., Holden, C. A., & Handelsman, D. J. (2005). Reproductive hormone reference intervals for healthy fertile young men: evaluation of automated platform assays. *The Journal of clinical endocrinology and metabolism, 90*(11), 5928–5936. https://doi.org/10.1210/jc.2005-0962
- [5] Barbotin, A. L., Ballot, C., Sigala, J., Ramdane, N., Duhamel, A., Marcelli, F., Rigot, J. M., Dewailly, D., Pigny, P., & Mitchell, V. (2015). The serum inhibin B concentration and reference ranges in normozoospermia. *European journal of endocrinology, 172*(6), 669–676. https://doi.org/10.1530/EJE-14-0932
- [6] Tajar, A., Forti, G., O'Neill, T. W., Lee, D. M., Silman, A. J., Finn, J. D., Bartfai, G., Boonen, S., Casanueva, F. F., Giwercman, A., Han, T. S., Kula, K., Labrie, F., Lean, M. E., Pendleton, N., Punab, M., Vanderschueren, D., Huhtaniemi, I. T., Wu, F. C., & EMAS Group (2010). Characteristics of secondary, primary, and compensated hypogonadism in aging men: evidence from the European Male Ageing Study. *The Journal of clinical endocrinology and metabolism, 95*(4), 1810–1818. https://doi.org/10.1210/jc.2009-1796

Supplementary Table S3. Detailed data about additionally dropped out patients from the study (N = 625) and control group (N = 57).

**CONTROL GROUP (N = 57)**		
**Factor or combination of factors**	**No of patients**	**Percentage (%) of patients**
Defined causal factors of male infertility [A] + Bitesticular volume <30 mL + Hormonal changes [B]	2	3.5%
Bitesticular volume <30 mL + Hormonal changes [B]	1	1.8%
Defined causal factors of male infertility [A] + Hormonal changes [A]	2	3.5%
Bitesticular volume <30 mL	3	5.3%
Hormonal changes [A]	45	78.9%
Defined causal factors of male infertility [A]	4	7.0%
**STUDY GROUP (N = 625)**		
**Factor or combination of factors**	**No of patients**	**Percentage (%) of patients**
Defined causal factors of male infertility [A] + Bitesticular volume <30 mL + Hormonal changes [B]	45	7,2%
Defined causal factors of male infertility [A] + Bitesticular volume <30 mL	13	2,1%
Defined causal factors of male infertility [A] + Hormonal changes [A]	55	8,8%
Bitesticular volume <30 mL + Hormonal changes [B]	31	5,0%
Hormonal changes [A]	368	58,9%
Bitesticular volume <30 mL	18	2,9%
Defined causal factors of male infertility [A]	95	15,2%

Annotations to Supplementary Table S3.

[A] Defined causal factors of male infertility: (1) genetic causes, (2) secondary hypogonadism, (3) congenital anomalies: systemic and/or in uro–genital tract, (4) serious sexual dysfunctions, (5) oncological diseases, (6) seminal tract obstruction, (7) other testicular factors (A. Acquired testicular damage (TD): Exposure to high dose radiation in Chernobyl; Testis trauma with volume change; Mumps orchitis; Orchitis, epididymitis; Testicular torsion; Hernia operation with ipsilateral TD; Epididymal cyst operation with ipsilateral TD; Hydrocele operation with ipsilateral TD; Other testis operation with ipsilateral TD; B. Secondary testicular damage: Anabolic steroids; Medication – salasopyrin, trexan; Status diagnosed post kidney transplantation)

[B] Hormonal changes: FSH ≥8.0 U/L and/or LH >9.4 U/L and/or FSH and/or LH <1.0 U/L and/or total testosterone <10.5 nmol/L

Supplementary Table S4. Impact of *C. trachomatis and M. genitalium* on blood parameters (selected infertile group only, after exclusion of patients for hormonal, testicular volume and infertility causal factors).

Parameter	STI negative n = 1345	*C. trachomatis* & *M. genitalium* positive together n = 30	STI negative *vs. C. trachomatis* & *M. genitalium* positive together, P-value [$$]	*C. trachomatis* positive n = 14	STI negative *vs. C. trachomatis* positive, P-value [$$, ①]	*M. genitalium* positive n = 16	STI negative *vs. M. genitalium* positive, P-value [$$, ①]
**PSA in serum, in *µg/L***	0.71 (0.12 – 5.43); 0.52; 0.99 [A]	0.79 (0.17 – 2.1); 0.60; 1.12	0.263	0.67 (0.19 – 2.1); 0.59; 1.19	0.148	0.89 (0.17 – 1.65); 0.67; 1.09	0.438

Annotations to Supplementary Table S4.

Data is presented as median (range), 25th and 75th centile.

Data is with non–parametric distribution.

[$$] p-value is calculated with Mann–Whitney test comparing two groups.

① Bonferroni correction of p–value for two tests

[A] Data accounting concentration of PSA in serum is not available for 52 STI negative patients.

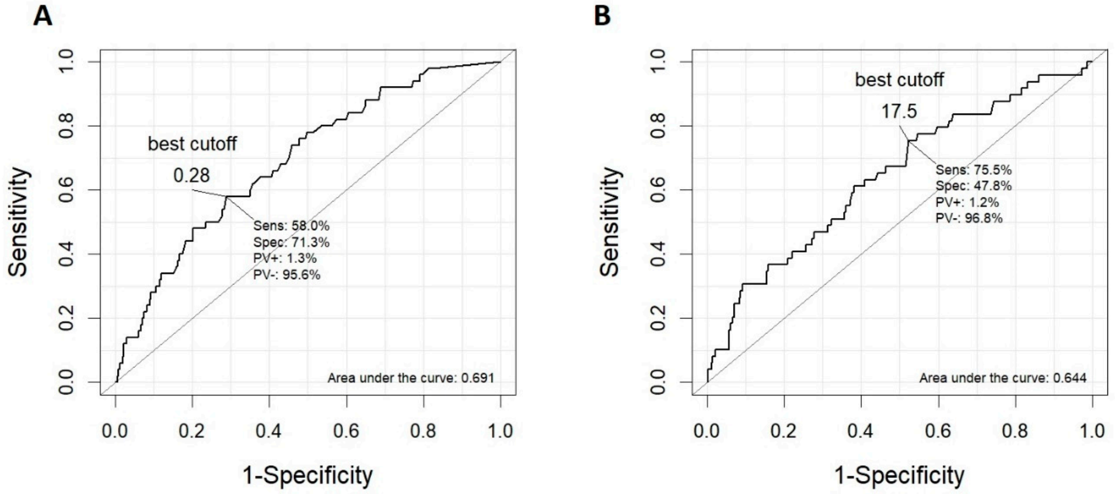

Supplementary Figure S1. Receiver operating curves for semen leucocytes concentration (A) and semen IL–6 concentration (B). Both unselected fertile and infertile group, without exclusion of patients for hormonal, testicular volume and infertility causal factors.

Annotations to Supplementary Figure S1.

Abbreviations: Sens – sensitivity, Spec – specificity, PV+ - positive prognostic value, PV- – negative prognostic value.
